# Acoustic Skyrmionic Mode Coupling and Transferring in a Chain of Subwavelength Metastructures

**DOI:** 10.1002/advs.202401370

**Published:** 2024-07-09

**Authors:** Wen‐Jun Sun, Nong Zhou, Wan‐Na Chen, Zong‐Qiang Sheng, Hong‐Wei Wu

**Affiliations:** ^1^ School of Mechanics and Photoelectric Physics Anhui University of Science and Technology Huainan 232001 China; ^2^ Center for Fundamental Physics Anhui University of Science and Technology Huainan 232001 China; ^3^ Institute of Energy Hefei Comprehensive National Science Center (Anhui Energy Laboratory) Hefei 230031 China

**Keywords:** acoustic metamaterials, mode hybridization, skyrmion

## Abstract

Skyrmions, a stable topological vectorial textures characteristic with skyrmionic number, hold promise for advanced applications in information storage and transmission. While the dynamic motion control of skyrmions has been realized with various techniques in magnetics and optics, the manipulation of acoustic skyrmion has not been done. Here, the propagation and control of acoustic skyrmion along a chain of metastructures are shown. In coupled acoustic resonators made with Archimedes spiral channel, the skyrmion hybridization is found giving rise to bonding and antibonding skyrmionic modes. Furthermore, it is experimentally observed that the skyrmionic mode of acoustic velocity field distribution can be robustly transferred covering a long distance and almost no distortion of the skyrmion textures in a chain of metastructures, even if a structure defect is introduced in the travel path. The proposed localized acoustic skyrmionic mode coupling and propagating is expected in future applications for manipulating acoustic information storage and transfer.

## Graphical abstract

The propagation and control of acoustic skyrmion along a chain of metastructures is proposed. The metastructure made with Archimedes spiral channel, the skyrmion hybridization is presented between neighboring structures. Then, it is experimentally observed that the skyrmionic mode of acoustic velocity field distribution can be robustly transferred covering a long distance, even if some structure defects are introduced in the travel path.

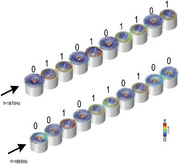

## 1 Introduction

Skyrmion, a prominent nontrivial topological texture characterized by a topological integer number, has aroused broad interest in different physical brunches, such as nucleons,^[^ ^1^ ^]^ Bose‐Einstein condensates,^[^ ^2^, ^3^ ^]^ magnetic materials,^[^ ^4^, ^5^, ^6^, ^7^ ^]^ and optics.^[^ ^8^, ^9^, ^10^, ^11^ ^]^ Particularly, in magnetic and optical systems, the skyrmion has grown into a large research field and discover various topological textures of skyrmionic modes: Néel type,^[^ ^12^, ^13^ ^]^ Bloch type,^[^ ^14^, ^15^ ^]^ anti‐type,^[^ ^16^, ^17^ ^]^ and so on. Recently, Skyrmion‐like vectorial textures are also constructed in various optical system for electric/magnetic fields ^[^ ^8^, ^9^, ^18^ ^]^ and spin ^[^ ^19^, ^20^ ^]^ configurations of photons. These rich topological vectorial textures give a promise for applications in information storage and data processing. Inspired by the development in magnetic materials and optical systems, there have been emerging interests in exploring similar skyrmionic textures in acoustic waves, even if the acoustic waves have long been regarded as spinless scalar waves. The updated knowledge for acoustic vectorial field ^[^ ^21^, ^22^, ^23^, ^24^, ^25^, ^26^ ^]^ give an avenue to discuss the acoustic topological texture, such as skyrmionic lattice for velocity vectorial distribution on a hexagonal acoustic metasurface,^[^ ^27^ ^]^ localized acoustic skyrmionic velocity fields on subwavelength acoustic metastructures,^[^ ^28^, ^29^ ^]^ and the acoustic skyrmionic spin textures in sound beam.^[^ ^30^ ^]^ In addition, phononic skyrmions have also been observed in phononic crystal based on elastic‐wave.^[^ ^31^ ^]^ The discovery of acoustic skyrmion induces novel topological mode in real space which has potential applications toward topologically robust ways to manipulate vectorial characteristics of the acoustic waves.

Acoustic skyrmion with nontrivial topological vectorial textures gives high promise for application of advanced data storage and robust transfer of acoustic information, while it remains extremely challenging for realizing motion control of the acoustic skyrmionic modes. As pioneer investigation, in magnetic materials, the motion control of the skyrmion is explored by external physical fields, such as temperature gradient,^[^ ^32^ ^]^ magnetic field gradient,^[^ ^33^ ^]^ electric current,^[^ ^34^ ^]^ curved racetracks,^[^ ^35^ ^]^ and the skyrmion Hall effect.^[^ ^36^ ^]^ The manipulation of magnetic skyrmion is crucial for development of advanced magnetic devices. In optical system, there are only few method for manipulation of the photonic skyrmion. A theoretical work on the manipulation of the skyrmion lattice shift by tuning the phase difference between sources.^[^ ^37^ ^]^ In experimental, a dynamic position control of photonic skyrmion is realized by imposing a phase profile with a spatial light modulator.^[^ ^38^ ^]^ However, to date, there are no report on the manipulation and transfer of the acoustic skyrmion textures.

In this work, we investigate the skyrmionic mode coupling between two acoustic metastructures made with Archimedes spiral channel, the skyrmion hybridization is found and giving rise to the skyrmionic mode splitting into bonding and antibonding skyrmionic modes. In this content, taking a step forward, we experimentally observe that the skyrmionic mode can be propagated and transferred covered a long distance along a chain of metastructures by the mode coupling, and almost no distortion of the skyrmion textures, even if a structure defect is introduced in propagating path. The robust transfer of the skyrmionic mode may be useful for advanced topological acoustic vectorial devices in applications of acoustic information processing and storage.

## 2 Acoustic Skyrmionic Mode Hybridization

**Figure 1:**
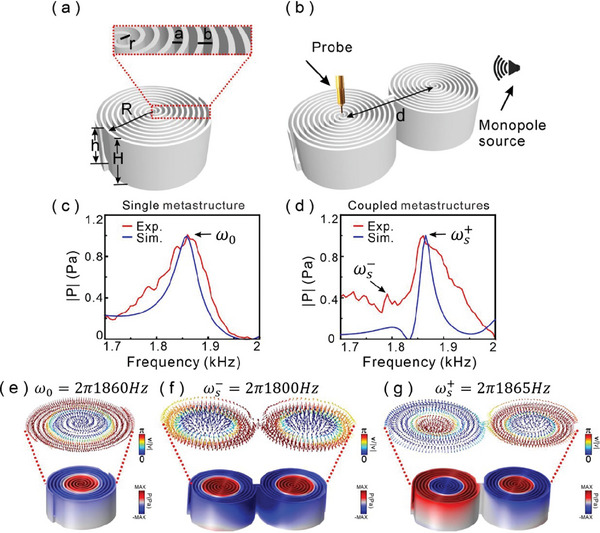
Schematic structure and acoustic pressure spectrum and skyrmionic mode hybridization. a) Single structure made with Archimedes spiral channel, the outer and inner radii are *R* and *r*, respectively, *b* is the spiral pitch, *a* is the channel width, *h* is channel height, and the whole structure height is H. b) Coupled metastructures with center‐to‐center as *d*. A monopole source and probe are used to obtain the pressure spectrum. Experimental (red) and simulated (blue) pressure spectra for c) single metastructure and for d) coupled metastructure. e) Simulated skyrmionic mode formed by the acoustic velocity field for the single structure; the direction of the acoustic velocity field is shown by the color arrows. f,g) Simulated skyrmionic hybridized modes for bonding mode and antibonding mode in coupled metastructures, respectively.

We implement the acoustic skyrmionic modes by using a 3D metastructure made with two Archimedean spiral channels as shown in Figure S1 (Supporting Information) sculpted in a cylinder, the Archimedean spiral channel can be described by the polar equation as ρ (θ) =  α + βθ, where $\beta \;=\frac{l}{2\pi }$ is the spiral growth rate, where α  =  0*mm*, *l*  =  10*mm*, the schematic diagram of the single structure is shown in **Figure** **1**a. The structure details are an outside radius *R*  =  50 *mm*, inner radius *r*  =  1*mm*, gap width *a*  =  3 *mm*, spiral pitch *b*  =  5 *mm*, structure height *H*  =  50*mm*, and the height of the Archimedes spiral channel is *h*  =  40*mm*. To investigate the skyrmionic mode coupling, we connect two metastructures with a straight channel, and the center‐to‐center distance is *d*  =  110*mm* as shown in Figure 1b. A monopole source marked by a speaker at the upper‐right position radiates the coupled metastructures, then we probe the pressure field at the metastructure surface to obtain the coupled mode distribution. Here, we show the symmetric‐configured structure to present hybridization of the two skyrmionic modes. In fact, the two coupled structures can also be designed as antisymmetric‐configured structure as shown in Figure S2 (Supporting Information). In order to study the skyrmionic mode coupling, we use the commercial software COMSOL Multiphysics to perform simulation calculations, in which a point source is placed 100 mm from the right structure and a probe is placed 5 mm above the center of the left structure to detect the pressure fields. The simulation results of the pressure spectra for the single and symmetric‐configured structures are shown in Figure 1c,d, respectively. We can find that the resonating frequency corresponded to the skyrmionic mode at ω_0_ =  2π 1860 *Hz* from the blue line in Figure 1c, which corresponds to the first‐order skyrmionic mode of the single structure. The red line corresponds to the experimental result which agrees well with the simulated result.

To demonstrate the first‐order skyrmionic mode, Figure 1e shows the pressure and velocity distributions supported on the well‐designed single spiral structure for the lowest order skyrmionic mode *m*  =  1, where the order number *m* describes the flipped number of velocity vectors along the radial direction. The colors in the structure mark the pressure field distributions, while the color arrows above the structure represent the configuration of the velocity fields. It can be seen from the vector orientation that the velocity field vector is upward at the single structure center, while the direction of the velocity field gradually flips with the increase of the radius and is finally directed downward at structure edge, which is a Néel‐type skyrmionic mode. The topological velocity texture is characterized by skyrmionic number as:

(1) $$S\;=\frac{1}{4\pi }\;\int \;\;\;\int dxdy\vec{n}\cdot \left(\partial_{x}\vec{n}\times \partial_{y}\vec{n}\right)$$

where $\vec{n}=\;Re\left(\vec{v}\right)/\left|Re(\vec{v})\right|$. The flipped number of the velocity field vectors $\vec{v}$ along the radius can be indicated by the skyrmionic number as *S*  =  1 for odd twists and *S*  =  0 for even twists.^[^ ^28^ ^]^ Here, we only show the lowest‐order Néel‐type skyrmionic mode for focusing the skyrmionic mode coupling, in fact, the single metastructure can support higher‐order skyrmionic modes with higher frequencies as described in ref. [28]

Next, we investigate the hybridization between two lowest‐order skyrmionic modes supported on the symmetric‐configured metastructure. By the numerical simulation, we find that the skyrmionic mode in single metastructure is split into a bonding mode $\omega_{s}^{-}=\;2\pi \;1800\;Hz$ with lower energy level and an antibonding mode $\omega_{s}^{+}=\;2\pi \;1865\;Hz$ with higher energy level due to the mode hybridization, as shown in blue line in Figure 1d. It is worth noting that the strength of the bonding mode is weaker than the antibonding mode, due to the near field the bonding mode with a larger mode volume resulting in lower mode strength in structure center by comparing the mode distributions in Figure 1f,g. Comparing the pressure field distributions of bonding mode and antibonding mode, we also can find that the bonding mode focuses on the structure center, while the antibonding mode homogenously distributes in each structure which is similar to the mode distribution of single structure, whichis why the peak of antibonding mode in pressure spectrum in closed to peak of single structure. We also experimentally measured the pressure field as a function of incident frequency as shown in red line, which agrees well with the simulated result, except that the difference in the peak width is due to the existence of the loss in the experiment. Furthermore, we also calculate the pressure spectra for different coupling distances from *d*  =  110–200mm as shown in Figure S2 (Supporting Information). The result indicates that the coupling strength decreases with increasing the distance, and the peak hybrid modes are close to each other. It means that the skyrmionic mode will be inhibited from propagating along the metastructure chain. Thus, in this work, we select coupling distance *d*  =  110mm to realize the propagation and manipulation of the acoustic skyrmionic modes.

To insight into the physical mechanism behind the skyrmionic mode coupling in two metastructures, we can calculate eigenfrequencies ω_0_, $\omega_{s}^{-}$ and $\omega_{s}^{+}$ of the single and coupling metastructures by the eigenfrequency solver of pressure acoustic module of the commercial software COMSOL Multiphysics, then the coupling factor can be expressed as $\kappa \;=\frac{\omega_{s}^{+}-\omega_{s}^{-}}{2\omega_{0}}$ which is dependent on the coupling distance *d*, thus the hybridized mode can be described with the coupled mode equations as^[^ ^39^, ^40^ ^]^:

(2) $$i\frac{d}{dt}\;\left(\begin{array}{c} a_{1}\\ a_{2} \end{array}\right)=\left(\begin{array}{cc} \omega_{0} & \kappa \omega_{0}\\ \kappa \omega_{0} & \omega_{0} \end{array}\right)\;\left(\begin{array}{c} a_{1}\\ a_{2} \end{array}\right)\;$$

where *a*_1_ and *a*_2_ denote the pressure field of the skyrmionic modes in two metastructures respectively. By solving the eigenvalue problem in the above Equation (2), the normalized orthogonal eigenvectors of [*a*_1_,*a*_2_]^T^ are [1, 1]^T^ and [− 1, 1]^T^, which correspond to the bonding mode and antibonding modes with the eigenfrequencies as $\omega_{s}^{-}=\omega_{0}\;-\kappa \omega_{0}$ and $\omega_{s}^{+}=\omega_{0}\;+\kappa \omega_{0}$, respectively. The coupling mode theory verifies the mode hybridization discussed in the aforementioned simulated and experimental results. The pressure and velocity field distributions of the bonding mode and antibonding mode are shown in Figure 1f,g. It is not different to find that not only the pressure field present mode hybridization, but also the skyrmionic textures of the velocity field have similar hybridized phenomena. Furthermore, the skyrmionic mode hybridization also be discussed for the antisymmetric‐configured metastructures in Figure S3 (Supporting Information)

## 3 Acoustic Skyrmionic Mode Transferring Along a Chain of Metastructures

**Figure 2:**
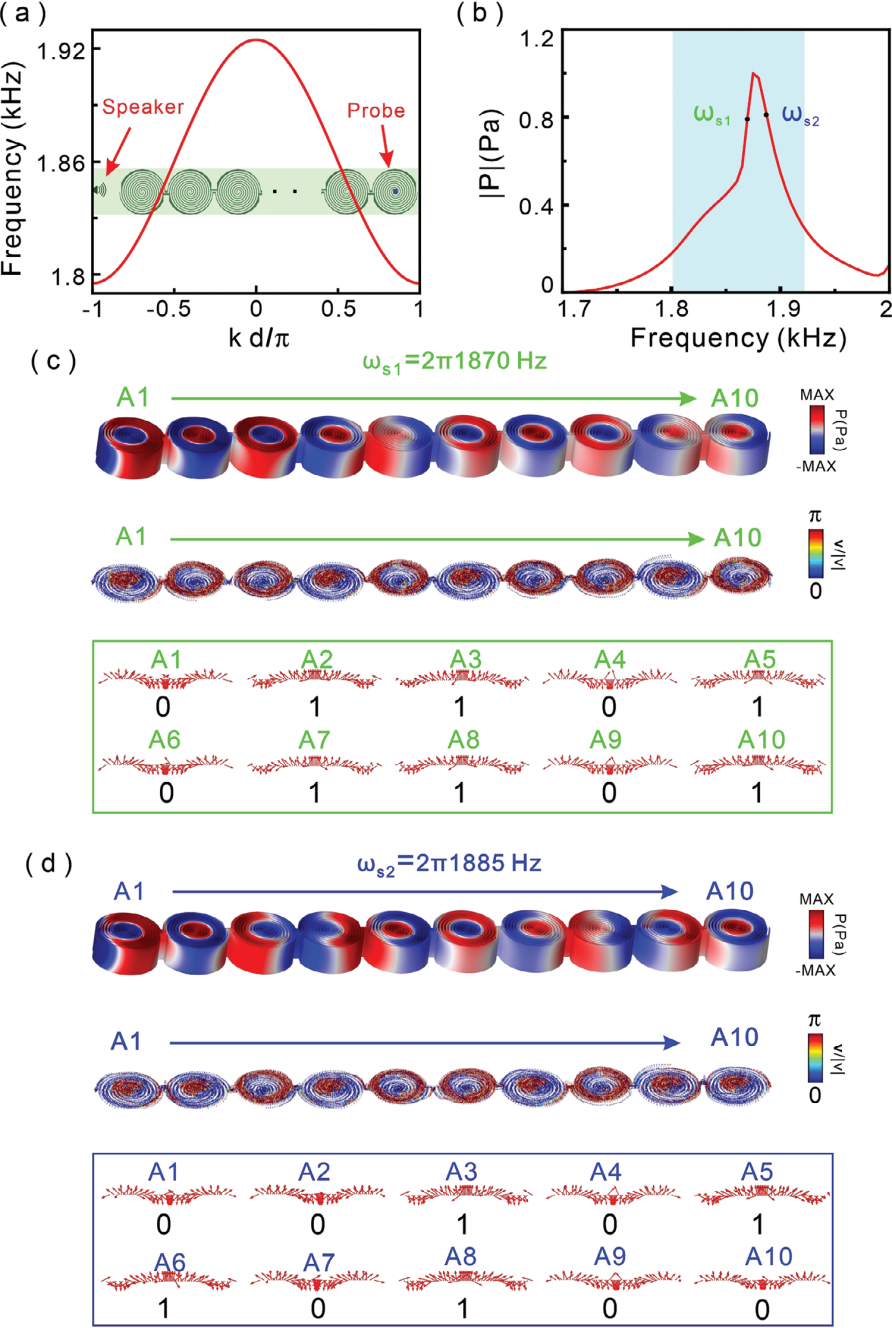
Acoustic skyrmionic mode transferring along a chain of metastructures. a) Intrinsic dispersion relation between the frequency and wave vector for infinite periodic structure by coupling mode theory, inset indicate the chain of metastructures. b) Sound pressure transmission spectra for ten metastructures in a chain. Sound pressure and velocity field distributions are shown in c) for frequency ω_s1_ and d) for frequency ω_s2_. The ten metastructures are numbered as “A1” to “A10”, and the skyrmionic modes are digitized as “0” and “1” according to upward and downward arrows at the structure center.

Next, we investigate the skyrmionic mode propagating along a chain of the metastructures as shown in the inset of **Figure** **2**a. Based on the coupling mode theory for infinite chains of symmetric‐configured metastructures, we can obtain the intrinsic dispersion relation between the frequency and wave vector as^[^ ^38^ ^]^ ω  = ω_0_ [1 + 2κcos(*kd*)], where *k* is the wave vector and *d* is the period of the chains of metastructures. The magnitude of the coupling strength κ and the sign affects the dispersive relation. Using the practical parameters ω_0_ =  2π 1860 *Hz*, κ  =  0.0174, *d*  =  110*mm*, then the dispersive relation as shown as red line in Figure 2a. From the results, we observe that the transmission band of the symmetric chain lies between the lower cutoff frequency of 1799 Hz and the upper cutoff frequency of 1921 Hz. To demonstrate the transmission properties of the proposed metastructure chains, without loss of generality, we constructed a chain with ten metastructures placed symmetrically, and an excited source is placed at the left side of the chain with 10 cm, the pressure field can be obtained by using a probe above the center of the tenth structure with 5 mm as shown as an inset of Figure 2a. We can find that the pressure spectrum has a transmission peak covering the frequency from 1.8 to 1.92 kHz as shown in red curve in Figure 2b. To observe the skyrmionic mode transport along the chain of metastructures, we pick two frequency ω_s1_ =  2π 1870*Hz* and ω_s2_ =  2π 1885*Hz* corresponding to high transmission, and we give the pressure and velocity field distributions in Figure 2c,d. The pressure and velocity field distributions also be experimentally measured and shown in Figures S4 and S5 (Supporting Information). The metastructures in the chain are marked as “A1” to “A10”. The velocity field vectors of skyrmionic mode are expressed by arrows, and they can be digitalized by defining the upward arrow at structure center as “1”, downward ones as “0”. We can find that the skyrmionic mode can be transported from the structure “A1” to “A10” along the chain for both frequency ω_s1_ and ω_s2_, but the arrow orders are different as shown in green and blue boxes with orders “01101 01101” and “00101 10100”, respectively. The skyrmionic modes propagating with digital orders give advanced information processing and transferring in a chain of metastructures. The skyrmionic modes transport in antisymmetric‐configured chain of the metastructures also be discussed in Figure S6 (Supporting Information), and the digital order of the skyrmionic modes in the chain as “01101 01101” for ω_s1_ =  2π 1870*Hz* and “00101 10100” for ω_s2_ =  2π 1885*Hz*.

**Figure 3:**
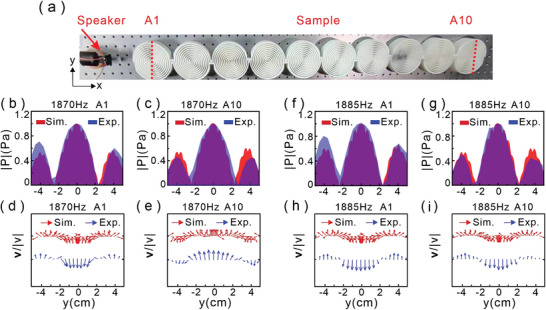
Experimental observation of the skyrmionic mode propagation. a) Schematic diagram of the experimental setup and sample. The red dashed line indicates the measured position at the first and tenth structure. b,c) Simulated (red area) and measured (blue area) pressure field distributions on structures “A1” and “A10” surface at frequency 1870*Hz*. Panels (d and e) show the corresponding simulated (red arrows) and measured (blue arrows) velocity field distributions along the red dashed lines in panel (a). For excited frequency as 1885*Hz*, panels (f–i) give the corresponding pressure field and velocity field distributions by simulation and experiment.

We have theoretically analyzed and numerically demonstrated that this structure can support Néel‐type skyrmionic mode transport above. Next, we experimentally observed the velocity vectorial distributions on the surface of the chain. As shown in **Figure** **3**a, we fabricated a sample based on the simulated structure using 3D printing technology and placed the loudspeaker as a point source 10 cm in front of the sample. At the beginning of the experiment, the sound signal generated by a signal generator (AFG1022, Tektronix) is amplified using the power amplifier (CPA2400, SinoCinetech) to drive the loudspeaker, and the sound pressure on the structure surface along the red dashed lines of the first and the tenth structures marked by “A1” and “A10” are measured by microphones. Figure 3b,c gives the pressure field distributions for the excitation frequency as ω_*s*1_ =  2 π 1870 Hz along the diameters of the structures “A1” and “A10”, respectively. We can find that the experimental measured (blue area) and numerical simulated (red area) results are consistent. Next, we use the measured pressure fields at different distances above the structure surfaces to derive the corresponding acoustic velocity field by $\;\vec{v}=\frac{1}{i\omega \rho_{0}}\;\nabla p$, where ω and ρ_0_ are the angular frequency and density of the air, respectively. Figure 3d,e shows the sound velocity field distribution of the Néel‐type skyrmionic modes, respectively. Here, the red arrows are simulated results, the blue arrows correspond to the experimental results. We can find that they are mutually consistent. Similarly, we also measure the acoustic pressure (as shown in Figure 3f,g) and velocity field (as shown in Figure 3h,i) distributions for the excitation frequency ω_*s*2_ =  2π 1885 Hz. The experimental results are agreement with our simulated results that the skyrmionic mode can be coupled and propagated from the first to the last one along the chain with the special digital orders.

**Figure 4:**
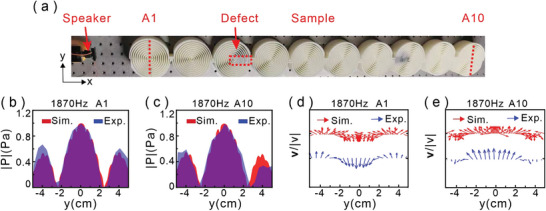
Experimental demonstration of the robustness of the skyrmionic mode propagation. a) Experimental setup and structure defect as shown in red dashed box. b,c) Simulated (red area) and measured (blue area) pressure field distributions along the red dashed lines in panel (a) for frequency 1870 Hz. d,e) Sound velocity field distributions along the red line on the structure by simulation (red arrow) and experiment (blue arrow).

To demonstrate the robust topological propagation of the skyrmionic modes along the metastructures, we intentionally introduced defects in the symmetric structures by inserting rigid materials to block the channels of the third structure as shown in red dashed box of **Figure** **4**a. Repeating the above experiment process, Figure 4b–e gives the experimental measured surface acoustic pressure and velocity field distributions of the first structure “A1” and the last one “A10” at the frequency ω_*d*1_ =  2π 1870 Hz for the breaking structure chain, which is identical to the simulation results. The experimental results indicate that the defects of the structure have no significant effect on the transfer of the skyrmionic modes.

Above, we have demonstrated that the acoustic skyrmionic mode can be propagated and transferred covered a long distance along a chain of metastructures by the hybrid mode coupling, and almost no distortion of the skyrmion textures, even if a structure defect is introduced in propagating path. In fact, the skyrmionic mode propagates along the metastructure chain depending on the coupling between adjacent metastructures, thus paths with different shapes can be constructed by the coupling metastructures to manipulating the acoustic skyrmionic mode transport for satisfying different applications. These results are unreported for acoustic skyrmion in previous works. However, as the skyrmions in magnetic materials and optical system, it is important to manipulate the skyrmionic mode transport for applications of sound information processing and storage.

## 4 Conclusion

In conclusion, we have proposed a concept of skyrmionic mode hybridization for symmetric‐configured and antisymmetric‐configured metastructures structures, the numerical simulation demonstrates the results based on the coupling mode theory, the skyrmionic mode will split into a bonding mode with lower level and an antibonding mode with higher level. In this context, we have constructed a chain of symmetric metastructures to support the skyrmionic mode propagating along the chain from the first to the last structure surface. The experimental results are in agreement with the simulated results that the skyrmionic mode with special digital orders to transfer the velocity field distributions for different frequencies. Lastly, we also demonstrate the robustness of the skyrmionic mode propagating along the chain by introducing the structure defect at the travel path. We believe that the acoustic skyrmionic mode coupling and propagating along the chain structure are useful for advanced topological acoustic vectorial devices in applications of acoustic information processing and storage.

## Conflict of Interest

The authors declare no conflict of interest.

## Supporting information

Supporting Information

## Data Availability

The data that support the findings of this study are available from the corresponding author upon reasonable request.
