# Supplementary material for: Acoustic Skyrmionic Mode Coupling and Transferring in a Chain of Subwavelength Metastructures
Source: Adv Sci (Weinh). 2024 Jul 9;11(34):2401370. doi: 10.1002/advs.202401370 (PMC11425862; doi:10.1002/advs.202401370)
Supplement: Supplementary file 1 — Supporting Information [file ADVS-11-2401370-s001.docx]

**Supplementary Materials**

Acoustic skyrmionic mode coupling and transferring in a chain of subwavelength metastructures

Wen-Jun Sun^1^, Nong Zhou^1^, Wan-Na Chen^1^, Zong-Qiang Sheng^1^ and Hong-Wei Wu^1, 2, 3*^

*^1^School of Mechanics and Photoelectric Physics, Anhui University of Science and Technology, Huainan 232001, China*

*^2^Center for Fundamental Physics, Anhui University of Science and Technology, Huainan 232001, China*

*^3^Institute of Energy, Hefei Comprehensive National Science Center (Anhui Energy Laboratory) Hefei 230031, China*

^*^Corresponding author: [hwwu@aust.edu.cn](mailto:hwwu@aust.edu.cn)

**Notes:**

1. **Schematic diagram of Archimedes spiral channel in single metastructure**
2. **Coupling strength between adjacent metastructures for different coupling distances *d***
3. **Skyrmionic mode hybridization of antisymmetric-configured metastructure**
4. **The experimental results of sound pressure and velocity field distributions for acoustic skyrmionic mode propagating from structure “A1” to “A10”**
5. **Acoustic skyrmionic modes propagation in antisymmetric-configured chain**
6. **Schematic diagram of Archimedes spiral channel in single metastructure**

**
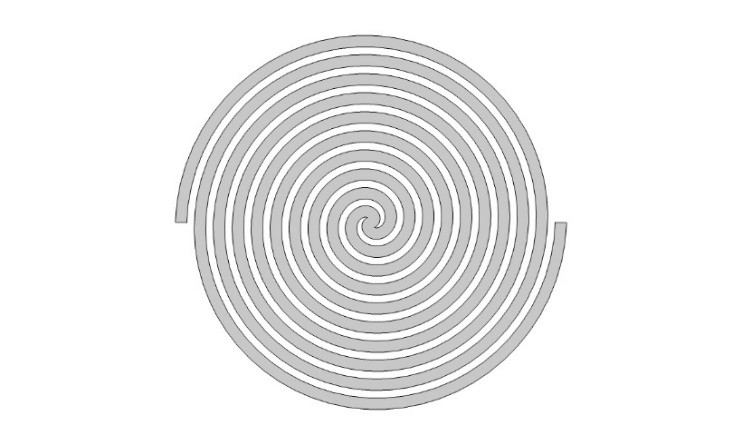
**

Fig. S1. Schematic diagram of Archimedes spiral channel as shown as the grey channel.

1. **Coupling strength between adjacent metastructures for different coupling distances *d***

Figure S2 shows the pressure spectra for different center-to-center distances between two adjacent metastructures. The result indicate that the coupling strength decreases with increasing the distance from *d* = 110mm to 200mm, and the peak hybrid modes close to each other. It means that the skyrmionic mode will be inhibited propagating along the metastructure chain due to the weak coupling.


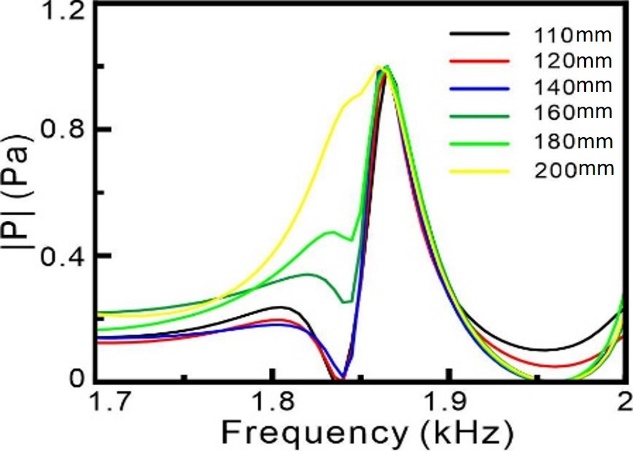


Fig. S2. The pressure spectra for different coupling distances from *d* = 110mm to 200mm.

1. **Skyrmionic mode hybridization of antisymmetric-configured metastructure**

To integrality, we also investigate Skyrmionic mode hybridization of antisymmetric-configured metastructures, as shown in Fig. S3(a). Figure S3(b) shows the pressure spectrum of the antisymmetric metastructure, and we observe that the coupling effect present like the results of the symmetric metastructure, splitting two resonance peaks $\omega_{a}^{-}=2\pi1800 Hz$ and $\omega_{a}^{+}=2\pi1865 Hz$, and the resonance spectra are similar to that of the symmetric structure. Next, we show the pressure field and velocity field distributions of the lowest-order Néel-type skyrmionic mode with resonant frequencies $\omega_{a}^{-}$ and $\omega_{a}^{+}$ in Figures S3(c) and S3(d), respectively. We can find that the results are very similar with ones of symmetric metastructure because acoustic wave is longitudinal wave.


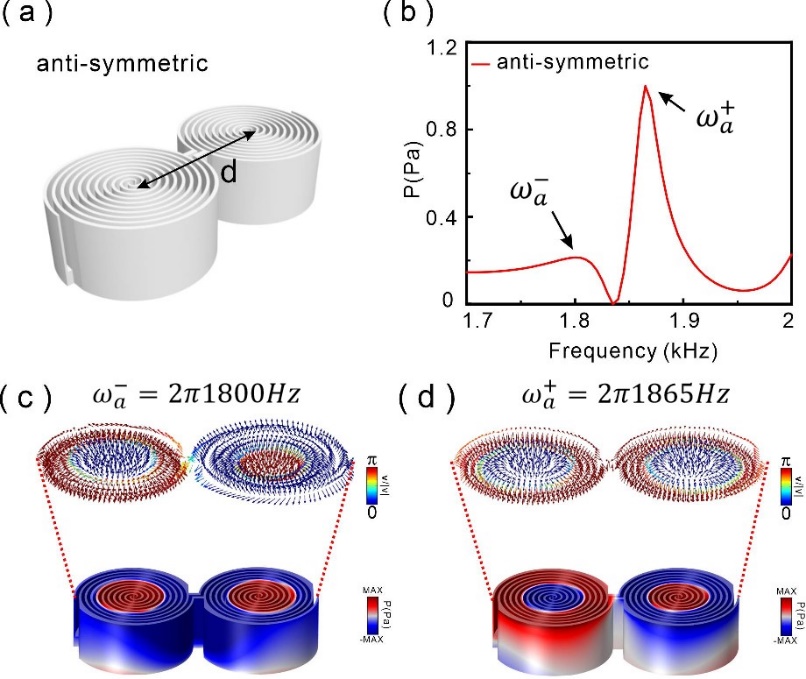


Fig S3. (a) Schematic diagram of the antisymmetric metastructure, the center distance between the two structures is d. (b) pressure field spectrum of antisymmetric metastructure. (c) and (d) The skyrmionic hybridized modes of the couple structure for bonding and antibonding modes, respectively.

1. **The experimental results of sound pressure and velocity field distributions for acoustic skyrmionic mode propagating from structure “A1” to “A10”**

In main text, we have calculated and suggest that the skyrmionic mode can be transported from the structure “A1” to “A10” along the chain for both frequency $\omega_{s1}$ and $\omega_{s2}$, but the arrow orders are different as shown in green and blue boxes with order “01101 01101” and “00101 10100” as shown in Figs. 2(c) and 2(d), respectively. To verify the results, we also practically measure sound pressure and velocity field distributions from structure “A1” to “A10” along the propagation direction at 1870Hz and 1885Hz as shown in Figs. S4 and S5.


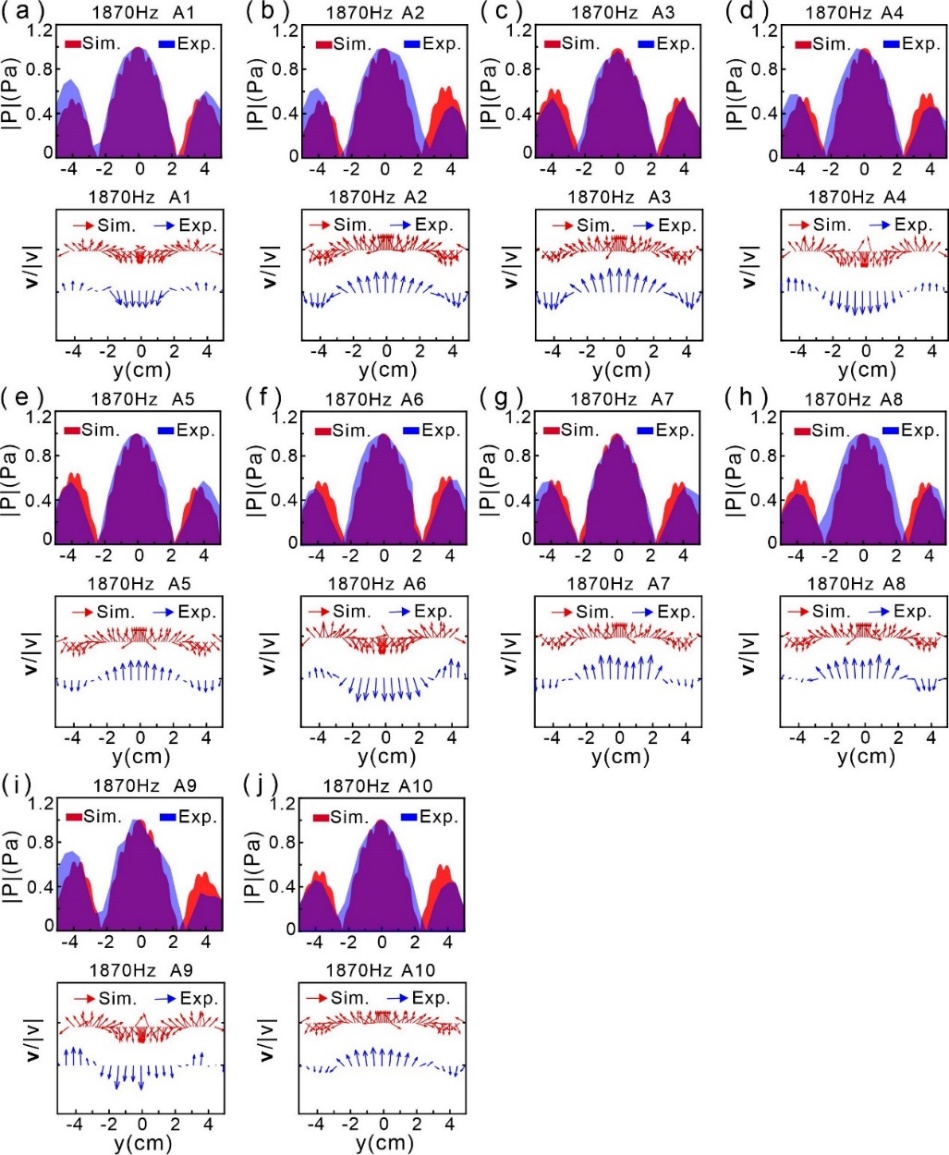


Fig. S4. The pressure and velocity field distribution by simulation and experiment along the sound propagation direction from “A1” to “A10” at frequency 1870Hz.


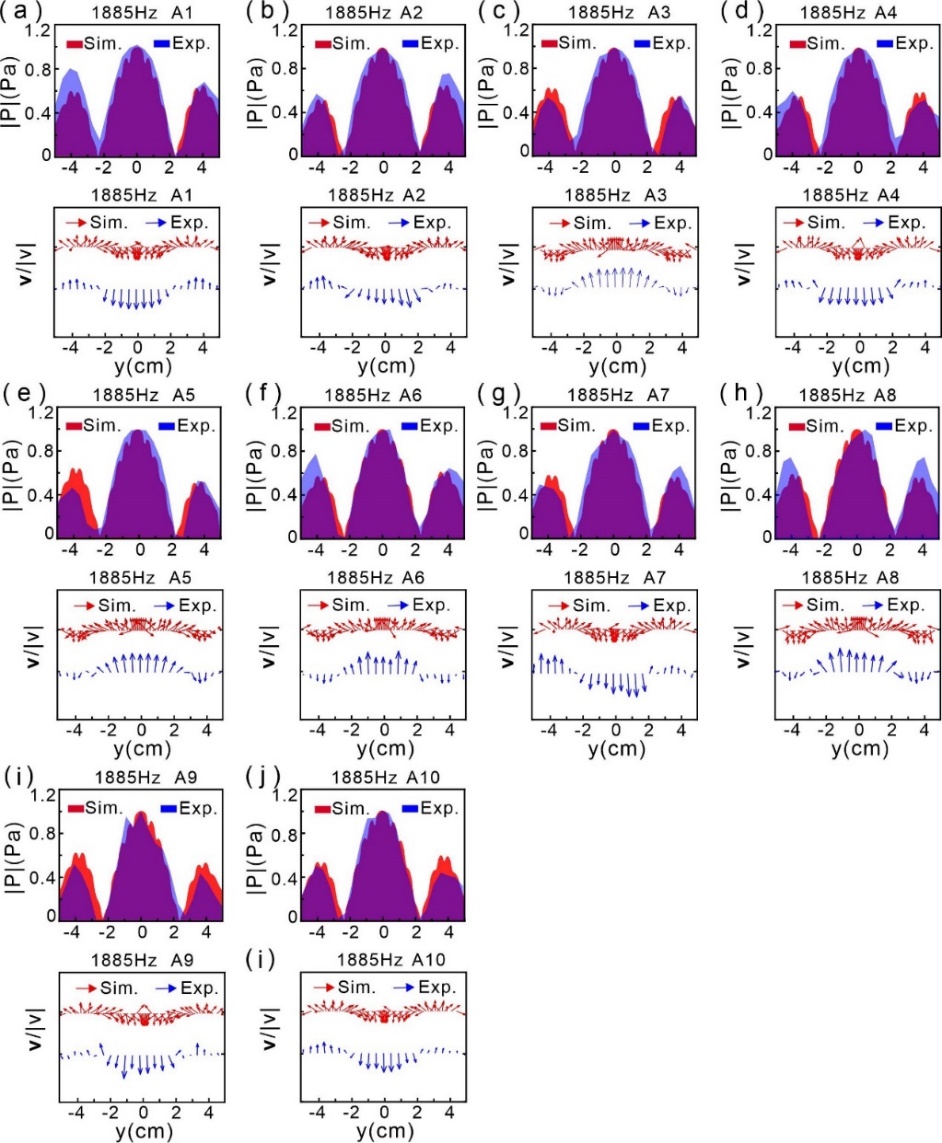


Fig. S5. The pressure and velocity field distribution by simulation and experiment along the sound propagation direction from “A1” to “A10” at frequency 1885Hz.

1. **Acoustic skyrmionic modes propagation in antisymmetric-configured chain**

As a supplementary, here, we also give the skyrmionic mode propagation along an antisymmetric metastructure chain. We use a monopole point source 10 cm next to the first structure to excite the skyrmionic modes, using a probe 5mm above the center of the last unit structure to obtain the transmission of the pressure field as show in Fig. S6(a). The chain of metastructures consisted with 10 structures as shown in Fig. S6(b). The simulated results indicate that an obvious transmission peak present at 1.875 kHz corresponding to the skyrmionic mode propagation. We select two frequency points with frequencies $\omega_{s1}$ and $\omega_{s2}$ to further verify the skyrmionic modes. As shown in Fig. S6(c) and S6(d), we give the acoustic velocity fields on the surfaces of the structures at the two selected frequency points, $\omega_{a1}=2\pi1870$ and $\omega_{a2}=2\pi1885$, which is Néel-type skyrmionic mode transmission as that of the symmetric structure.


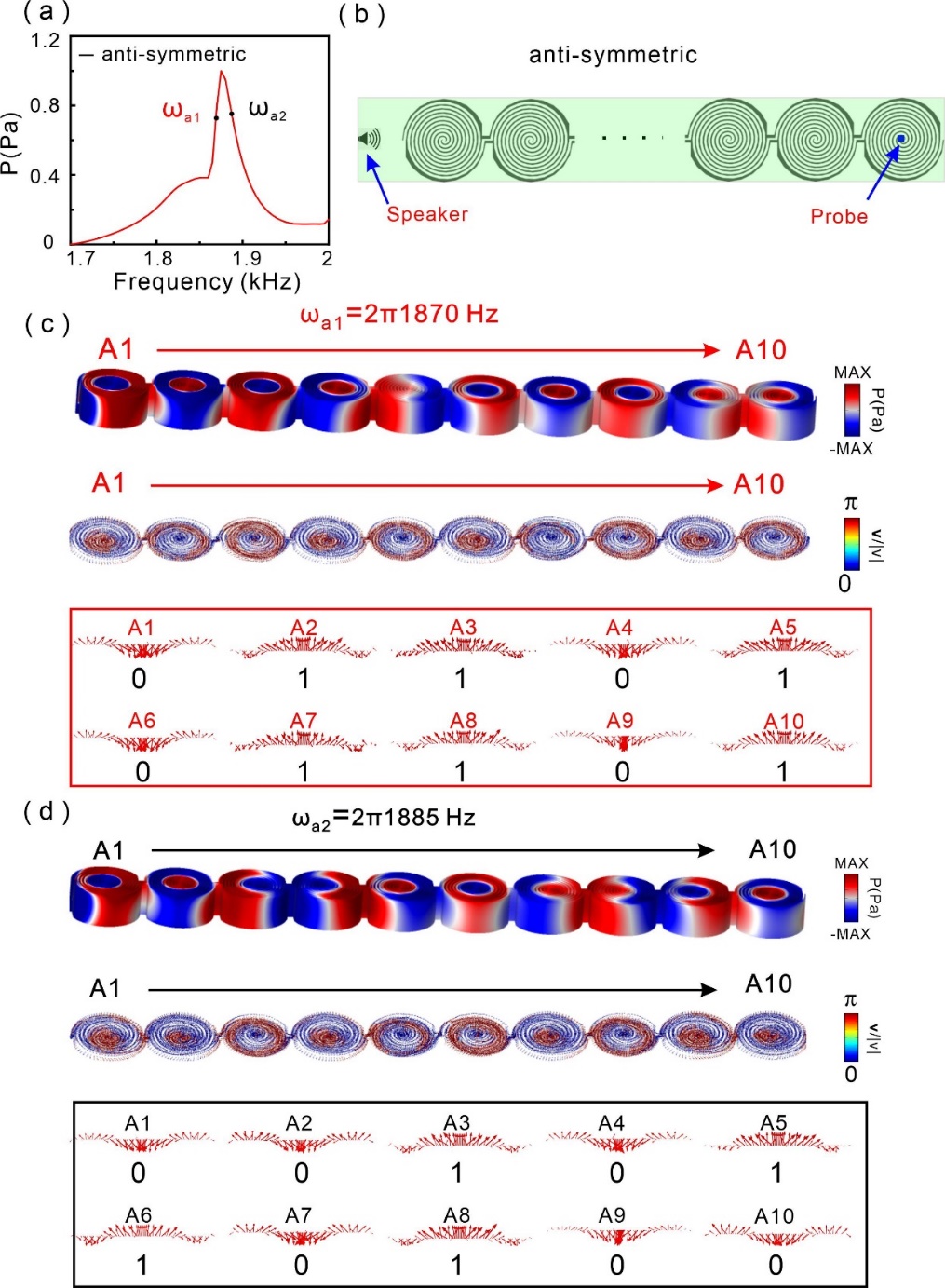


Fig S6. Simulation results (a) Anti-symmetric sound pressure spectra. (b) anti-symmetric structure chains. Sound pressure and velocity field distributions are shown in (c) for frequency $\omega_{a1}$ and (d) for frequency$\omega_{a2}$. The ten metastructures are numbered as “A1” to “A10”, the skyrmionic mode are digitized as “0” and “1” according to upward and downward arrows at the structure center.
